# Revisiting Ki-67 Assessment in Canine Mast Cell Tumours: From Manual Hotspot to Automated Global Analysis

**DOI:** 10.3390/vetsci13020198

**Published:** 2026-02-18

**Authors:** Rebeca Scalco, Elena Wasmer, Kathrin Jäger, Sven Rottenberg, Heike Aupperle-Lellbach, Simone de Brot

**Affiliations:** 1 Institute of Animal Pathology, University of Bern, 3012 Bern, Switzerland; 2 LABOKLIN GmbH & Co. KG, 97688 Bad Kissingen, Germany; 3 Bern Center for Precision Medicine, University of Bern and Inselspital, 3008 Bern, Switzerland

**Keywords:** digital pathology, Ki-67, mast cell tumour, canine

## Abstract

Canine mast cell tumours (MCTs) show highly variable behaviour, and Ki-67 is an established prognostic indicator. Conventional Ki-67 assessment is manual and restricted to small hotspot areas, limiting reliability. This study presents a semi-automated whole-tumour tissue section (global) Ki-67 analysis workflow, outlines its limitations, and examines correlations with hotspot counts and clinical outcome. A total of 309 canine MCTs were assessed using a deep-learning-assisted quantification with commercial software. Global Ki-67 metrics were correlated with hotspot Ki-67 counts and histomorphologic tumour grades, as supported by clinical follow-up data from 68 dogs. The defined analytic workflow enabled an overall feasible global Ki-67 assessment in canine MCTs. The region-of-interest (ROI) definition required frequent manual adjustments, whereas Ki-67 quantification was fully automated and rapid. Global Ki-67 metrics correlated with manual hotspot counts, with Ki-67-positive cell density on average twice as high in tumour hotspots compared with whole tumour sections, with differences ranging up to 38-fold. Exploratory survival analyses suggested promising predictive power, warranting validation in a robust survival study. With established digital pathology tools, global whole-tumour assessment of Ki-67 and other biomarkers is feasible. It should become the new standard for defining robust prognostic and predictive markers in canine mast cell and other tumours.

## Simple Summary

Canine mast cell tumours (MCTs) show highly variable behaviour, making it difficult to predict outcomes. Ki-67 is a protein that marks all proliferating cells and is commonly used as a prognostic indicator. Traditionally, Ki-67 is measured manually in small “hotspot” areas of the tumour, but results can vary between pathologists, limiting reproducibility. This study developed a semi-automated workflow to measure Ki-67 across the whole tumour tissue section using digital pathology and deep-learning tools. A total of 309 tumours were analysed, and the results were compared with manual hotspot counts, tumour grade, and clinical outcome. The workflow enabled rapid and reliable Ki-67 measurement across a range of hundreds of thousands to millions of cells, though defining the tumour region often required some manual correction. Global Ki-67 levels strongly correlated with manual hotspot counts and showed promising potential to predict survival, though larger studies are needed. Importantly, this approach can be applied to Ki-67 and other biomarkers, providing a standardized, reproducible, and comprehensive method for assessing tumour behaviour and improving prognostic and treatment decisions in veterinary oncology.

## 1. Introduction

Mast cell tumours (MCTs) are one of the most common neoplasms in dogs, accounting for 16–21% of all canine skin tumours [1]. The biological behaviour of these neoplasms is highly variable, ranging from benign tumours cured by surgery to malignant tumours with aggressive growth and a high metastatic rate, making prognosis challenging [2,3,4]. At present, prognostic assessment currently relies mainly on the established Patnaik three-tier and Kiupel two-tier histomorphologic grading systems. In the original Patnaik study from 1984, 93% of dogs with grade I MCT survived long term, compared with 44% with grade II and 6% (1/17) with grade III tumours [5]. The more recent grading system of Kiupel simplifies grading into high- and low-grade categories, with a median survival time of less than 4 months for high-grade MCTs but more than 2 years for low-grade MCTs [6]. Beyond tumour histomorphology, cell proliferation markers enhance prognostic accuracy, specifically argyrophilic nucleolus organizer regions (AgNORs), Ki-67, and the mitotic count. Ki-67 is a nonhistone nuclear protein expressed only in dividing cells [7]. Ki-67 is, therefore, a reliable marker for evaluating the proliferative status of normal and abnormal cell populations. Since its initial discovery, several further antibodies, including MIB-1 (Molecular Immunology Borstel), have been developed to detect the Ki-67 protein by IHC [8]. Traditionally, nuclear Ki-67 expression is defined as either negative or positive, without consideration of staining intensity or nuclear staining pattern. However, growing evidence indicates that both staining intensity and nuclear pattern are prognostically relevant, may provide additional insights into tumour biology, and therefore, warrant consideration in the evaluation of Ki-67 [9,10,11,12]. In canine MCTs, Ki-67 is an established prognostic marker [13,14,15,16,17,18] that is ideally combined with other markers, such as histologic grading, c-kit mutations, and KIT IHC staining patterns, to improve prognostic accuracy [19].

Two significant challenges in evaluating Ki-67 and establishing reliable prognostic cut-off values in canine MCTs are the lack of standardized evaluation methods and the limitation of conventional assessments to a small, hotspot-restricted area. There is an urgent need to standardize the selection and definition of the analysed tumour area (hotspot versus whole tumour section), to ensure accurate tumour and cell segmentation with exclusion of non-neoplastic elements, to establish clear criteria for cellular positivity, to use consistent manual or automated counting methods, and to provide robust quantitative outputs. At present, Ki-67 in canine MCTs is typically assessed according to the definition by Webster et al. in 2007 [14]. Specifically, Ki-67 positive nuclei are counted within a small (0.3125 mm^2^) region exhibiting the highest density of Ki-67-positive cells (the hotspot), with both hotspot selection and cell counting performed manually and by light microscopy. Based on this approach, Webster et al. [14] proposed a prognostic threshold of 23 Ki-67-positive cells per assessed hotspot, above which shorter survival times were observed. However, considerable confusion exists in the literature regarding the exact dimensions and selection of the evaluated area in this method, and significant interobserver variation is well known. These ambiguities, together with the limitations outlined above, reduce the reliability and comparability of this assessment approach.

In contrast to absolute Ki-67-positive cell enumeration, several investigations have quantified Ki-67 expression as a relative measure, calculating the proportion of Ki-67-positive mast cells among total tumour cells and expressing this value as a Ki-67 proliferation index (PI) [18]. Using this approach, a Ki-67 PI threshold of 1.8% was shown to be strongly associated with survival in dogs with intermediate-grade MCTs [15]. Berlato et al. confirmed that Ki-67 PI has prognostic significance independently of the mitotic index in canine MCT [20]. These findings indicate that both absolute and relative Ki-67 metrics can convey prognostic information and should be considered when defining clinically meaningful thresholds. However, relative Ki-67 proliferation indices are critically dependent on the accurate identification of neoplastic cells within the evaluated area, because substantial admixture of Ki-67-negative non-tumour cells can lead to underestimation of true tumour proliferative activity. In particular, the presence of abundant reactive stromal and inflammatory cells—many of which may themselves express Ki-67 to varying extents—remains a major confounding factor for reliable, tumour-specific Ki-67 analysis.

Recent advances in digital pathology have enabled whole-slide imaging (WSI) and automated image analysis, facilitating comprehensive assessment of tissue biomarkers across entire tumour tissue sections rather than small, sampled areas [21,22]. Global assessment theoretically addresses tumour heterogeneity more comprehensively and reduces sampling bias inherent to hotspot selection, whilst deep-learning-based image analysis offers potential for improved reproducibility and objectivity.

This study aimed to: (i) develop and validate a semi-automated workflow for global Ki-67 assessment in canine MCTs using digital pathology and deep-learning-assisted image analysis; (ii) characterise the technical requirements, feasibility, and limitations of this approach; (iii) evaluate correlations between global Ki-67 metrics and manual hotspot counts and histologic tumour grade and (iv) perform an exploratory survival analysis to assess the prognostic potential of global Ki-67 metrics.

## 2. Materials and Methods

### 2.1. Case Selection and Information

In this retrospective study, the cohort comprised 309 cases of canine cutaneous mast cell tumour (MCT) biopsies submitted for tumour diagnostics between 2012 and 2023 to Laboklin GmbH & Co. KG, Bad Kissingen, Germany, and the Institute of Animal Pathology, University of Bern, Bern, Switzerland. All cases corresponded to initial diagnoses, and no prior treatments, such as chemotherapy, were documented. The case inclusion criteria required a confirmed histopathologic diagnosis by a board-certified veterinary pathologist and the availability of a Ki-67 immunohistochemically (IHC) stained tumour tissue glass slide or a formalin-fixed, paraffin-embedded tumour tissue block for subsequent Ki-67 staining. The available case-related information included age at biopsy, sex, neutering status, and breed. In addition, manual assessment results, i.e., tumour grading by Patnaik [5] and Kiupel [6], and Ki-67 tumour hotspot counts were available for 242 of 309 cases. Of these, 68 cases had both tumour grading and exact Ki-67 hotspot counts recorded, as they were investigated in greater molecular detail and have been reported previously [23]. The remaining 178 cases originated from the diagnostic tissue archive, lacked tumour grading, and were reported using a binary Ki-67 classification (≤23 vs. >23 Ki-67-positive cells per hotspot area, as defined by Webster et al. [14]). Binary Ki-67 assessment is routinely preferred over exact hotspot quantification in diagnostic practice due to its greater time efficiency. Clinical outcomes were available for 68 of 309 cases, approximately half of which originated at the Swiss institution. Inclusion of cases from this institution was based primarily on the availability of follow-up data, which was essential for the outcome-related exploratory analyses conducted in this study (Supplementary Table S1).

### 2.2. Ki-67 Immunohistochemistry (IHC)

Due to the retrospective nature of the study and differences in institutional protocols, immunohistochemical staining procedures varied between the two case series, as detailed below. A detailed comparison of all staining protocol parameters is provided in Supplementary Table S2.

#### 2.2.1. Case Series Germany

The majority (248/309; 80%) of the investigated cases were submitted to Laboklin GmbH & Co. KG (Bad Kissingen, Germany), where IHC staining was performed according to the following protocol. Formalin-fixed and paraffin-embedded (FFPE) tissue sections (2 µm) were mounted on coated slides, dried overnight at 58 °C, and processed for Ki-67 expression. Antigen retrieval was performed in EDTA buffer (Zytomed Systems GmbH; Berlin, Germany) (pH 9.0) at 96 °C for 25 min in a commercial steam heater. Sections were incubated for 60 min at room temperature with monoclonal anti-mouse MIB-1 antibody (Dako, #M7240; dilution 1:200). Negative controls were prepared by replacing the primary antibody with buffer, and canine lymph node tissue served as a positive control. Immunoreactivity was visualized using the ZytoChem Plus HRP Polymer Kit (Zytomed Systems GmbH; Berlin, Germany) with DAB as chromogen, followed by counterstaining with Mayer’s haematoxylin.

#### 2.2.2. Case Series Switzerland

The remaining 61/309 (20%) cases were submitted to the Institute of Animal Pathology at the University of Bern (Switzerland), where IHC staining was performed according to the following protocol. FFPE tissue sections (2–3 µm) were mounted on positively charged slides (Color Frosted Plus, Biosystems, Muttenz, Switzerland), dried for 35 min at 60 °C, and dewaxed using Bond Dewax solution (Leica Biosystems). All subsequent steps were performed on Bond-III immunostainers (Leica Biosystems, Melbourne, Australia). Heat-induced epitope retrieval was performed using Bond epitope retrieval solution 2 (pH 9, Leica Biosystems) for 20 min at 95 °C. To reduce nonspecific binding, a protein-blocking solution was applied for 10 min at room temperature, as was conducted for all subsequent incubations. Slides were then incubated with the primary monoclonal anti-mouse MIB-1 antibody for 15 min. (Dako, #M7240; dilution 1:50). Detection was performed using the Bond Polymer Refine Detection Kit (Leica Biosystems). Endogenous peroxidase activity was blocked for 5 min, followed by incubation with the secondary antibody for 8 min and the peroxidase-labelled polymer for 8 min. Both the secondary antibody and polymer reagents were supplemented with 2% dog serum (LabForce, Nunningen, Switzerland) to further reduce nonspecific binding. Chromogenic development was achieved using 3,3′-diaminobenzidine (DAB)/H_2_O_2_ for 10 min. Slides were counterstained with haematoxylin and mounted. Negative controls were processed in parallel by replacing the primary antibody with wash buffer. External (human epidermis) and internal (adjacent canine epidermis) positive controls were included for each run.

### 2.3. Digital Ki-67 Analysis

**Figure 1:**
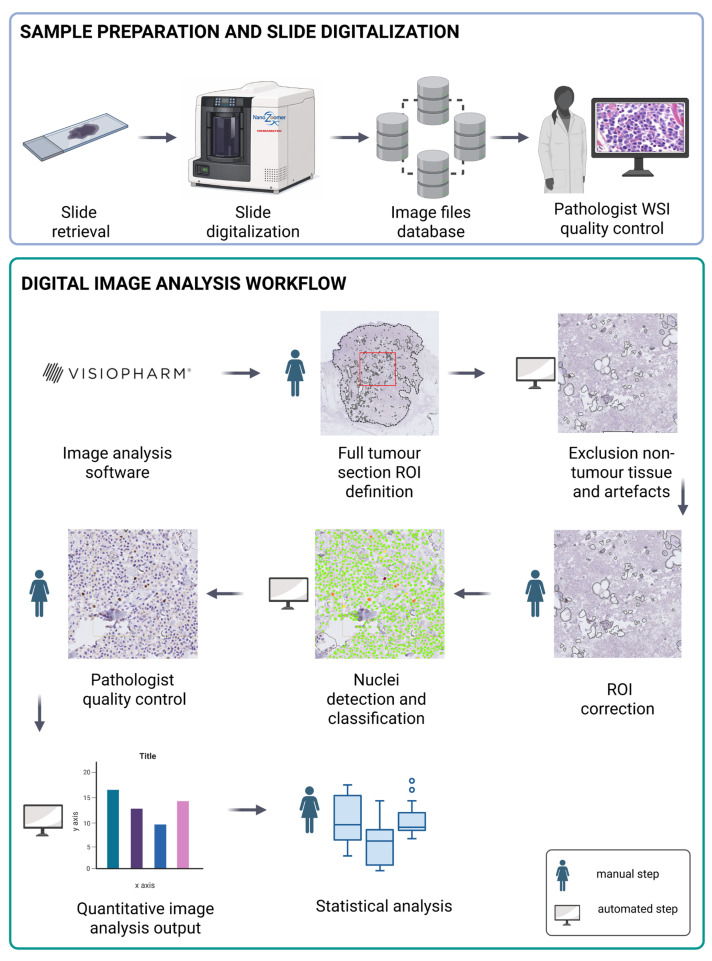
Comprehensive workflow for digital Ki-67 quantification in tumour tissue sections. The process begins with slide preparation and whole slide imaging (WSI), during which slides are digitized and undergo pathologist-quality control. The digital image analysis workflow utilizes Visiopharm software. Pathologists define the tumour outline as a region of interest (ROI—red square) encompassing the entire tumour tissue section, followed by automated exclusion of non-tumour tissue and artifacts, with manual ROI correction where required. Automated nuclei detection and classification algorithms identify Ki-67-positive and negative cells, with subsequent pathologist quality control to validate the automated quantification (nuclear labelling: green: negative; yellow: weak; orange: moderate; dark red: strong Ki-67 positive). The workflow generates quantitative image analysis outputs that feed into statistical analysis for comparative studies. Manual steps performed by trained pathologists (person icons) are integrated with automated computational processes (computer icons) to ensure accurate and reproducible tumour cell proliferation measurements. The figure was created with BioRender.com.

All Ki-67-stained tumour tissue slides were scanned for the subsequent digital evaluation using the Aperio AT2 Scanner (Leica Biosystems, Deer Park, IL, USA) and the Hamamatsu NanoZoomer S360MD Slide Scanner System (Hamamatsu Photonics, Shizuoka, Japan) at 40× magnification, corresponding to a spatial resolution of 0.23 µm/pixel. While different slide scanners and immunohistochemical staining protocols can affect digital slide analysis, Ki-67 expression was first assessed and compared between the two IHC series to ensure that the analysis was not affected by protocol-dependent differences. To this end, brown DAB staining was quantified on original whole-slide images by calculating the mean pixel intensity of the HDAB–DAB feature within the defined tumor area for each slide. Pixel intensity thresholds for weak, moderate, and intense staining were defined by direct slide review (i.e., visual inspection while displaying only the HDAB–DAB feature); the applied thresholds are specified in the Ki-67 analysis section. Pixel values ranged from 25 (dark brown) to 255 (white), with mean nuclear values of 89 (both IHC protocols), 65 (64 for the German protocol, 66 for the Swiss protocol), and 39 (38 for the German protocol, 40 for the Swiss protocol) corresponding to weak, moderate, and strong DAB positivity, respectively. Comparison of the mean HDAB–DAB pixel intensities of all Ki-67-positive nuclei, pooled across slides, between the two IHC series revealed a statistically significant inter-laboratory difference of approximately 2 pixel intensity units (*p* = 0.00001), as determined by nonparametric testing (Wilcoxon rank-sum test). However, this difference corresponds to <10% of the separation between adjacent staining intensity categories (weak, moderate, strong) and to <1% of the full dynamic range of DAB staining intensity. Therefore, the observed difference does not affect staining intensity classification or Ki-67 expression interpretation, confirming the practical equivalence of the two IHC staining protocols. Accordingly, all slides were analysed digitally using an identical workflow, and results were not stratified by scanner or staining protocol. Ki-67 IHC expression was assessed across the full tumour section using automated digital analysis of whole slide images (WSI) with Visiopharm software (version 2024.09 ×64, Visiopharm, Hørsholm, Denmark). The analysis workflow involved the following sequential steps: (I) manual delineation of the tumour outline, which was defined as the region of interest (ROI); (II) detection of tissue and staining artifacts and non-neoplastic tissue within the ROI and subsequent clearance from the primary tumour ROI; (III) detection and classification of individual cell nuclei; (IV) output generation and export; (V) quality control and scoring of each analysed slide for defined criteria. The full workflow of the semi-automated digital global tumour Ki-67 assessment is described and illustrated in Figure 1.

#### 2.3.1. Definition of the Region of Interest (ROI)

The ROI for Ki-67 analysis comprised all available tumour tissue on the respective whole-slide image and was delineated as follows: The tumour outline was defined manually due to the very heterogeneous presentation of the studied tumours regarding tissue sectioning and staining quality, tumour histomorphology, and the definition of the tumour boundary relative to surrounding healthy tissue. The heterogeneous tumour presentation limited the options for designing and running a fully automated tumour detection program. Ulcerated areas were excluded. In tumours with significant peritumour inflammatory reaction, the tumour outline was less well-defined and often required slide revision at higher magnification. The time for manual ROI definition (i.e., drawing of the tumour outline) per slide was overall low, ranging from less than 1 min to 5 min, depending on tumour size and the definition of the tumour border. The next step was to exclude any non-tumour tissue (i.e., adnexa, medium to large-sized blood vessels, adipose tissue, lymphoid follicles) and artefacts within the ROI. For this purpose, a deep learning (DL) classifier (U-Net) was trained on 50 representative annotated slides from all three series for more than 1,000,000 iterations (input magnification 5×) and subsequently applied to detect structures to be excluded from the ROI. Manual corrections were frequently necessary and often extensive due to the highly heterogeneous morphology of the different tissue sections and the presence of tissue artefacts. As a result, ROI refinement represented the most time-consuming step in the workflow. The automated program needed only 0.5 to 2 min per slide and could be run in the background as batch processing without interrupting the workflow. In contrast, manual corrections were relatively labour-intensive, taking up to half an hour per slide in cases with many tissue artefacts and embedded non-neoplastic tissue, such as adnexal structures. Overall, approximately 1 in 10 tissue slides required substantial manual amendments, defined as corrections that took more than 5 min. Each slide was evaluated independently by one junior and one senior pathologist to determine whether manual amendments were necessary and, when applicable, to assess the quality of the tissue segmentation after correction.

#### 2.3.2. Ki-67 Analysis and Result Export

**Figure 2:**
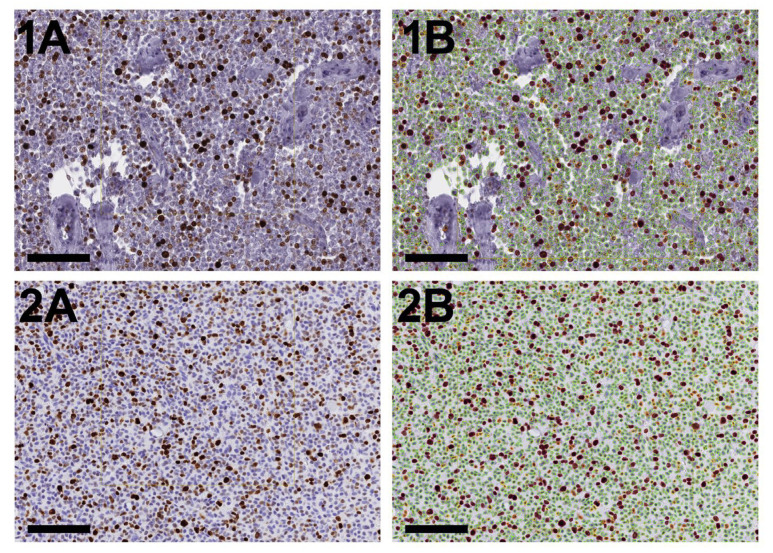
Nuclear immunohistochemical (IHC) Ki-67 expression in canine mast cell tumours shows evident staining heterogeneity with comparable staining quality between the two IHC protocols used. Representative cases illustrate Ki-67 IHC staining performed using two different protocols. (**1A**) Ki-67 staining based on the protocol from the laboratory) in Germany; (**1B**) the same tumour region as shown in (**1A**) with digital labelling of individual nuclei classified as negative (green), weak (yellow), moderate (orange), and intense (dark red) Ki-67 positive; (**2A**) Ki-67 staining based on the protocol from the laboratory in Switzerland; (**2B**) the same tumour region as shown in (**2A**) with digital labelling of individual nuclei classified as indicated for (**1B**). Scale bar = 100 µm.

Once the ROI has been defined, an automated nuclear detection and classification program was run in the background (batch processing). Specifically, the predesigned Visiopharm APP #10170, “IHC, Nuclei Detection, AI,” was used, which is based on DL classification (U-Net) with 100K iterations. With this APP, all nuclei within the defined ROI were detected, without discrimination between neoplastic and non-neoplastic cells. In all cases, nuclear Ki-67 staining intensity varied markedly within individual tumours (Figure 2). Although the biological and clinical relevance of Ki-67 expression intensity remains unclear, it was considered necessary to record it. Accordingly, in addition to classification as positive or negative, Ki-67-positive cells were further categorized by staining intensity. Nuclei were classified as Ki-67-negative, weakly, moderately, or strongly positive based on mean HDAB–DAB pixel values measured per nucleus. Pixel values were extracted from the central 25–75% of each nuclear object to reduce edge artefacts and background influence. Because HDAB–DAB pixel values range from 0 (strongest DAB labelling) to 255 (no labelling), values were interpreted inversely with staining intensity. Accordingly, nuclei were classified as negative (>170), weak (120–170), moderate (70–120), or strong (<70) based on mean HDAB–DAB pixel values. For each slide, the following output was defined: Assessed tumour tissue area (mm^2^); total counts of Ki-67-negative, weak, moderate, and strong nuclei, with calculation of total Ki-67-positive counts; calculation of H-score (defined as (1 × percentage of weakly stained nuclei) + (2 × percentage of moderately stained nuclei) + (3 × percentage of strongly stained nuclei), ranging from 0 to 300); the density of Ki-67-positive and strongly positive nuclei was calculated as the number of respective nuclei per mm^2^ of tumour tissue. The H-score was calculated because it provides a standardized, semi-quantitative measure that integrates staining intensity and the proportion of positive cells, enabling a more nuanced and digitally applicable assessment than percentage positivity alone [24,25]. Strongly Ki-67-positive nuclei were analysed as a separate category because, upon review of labelled sections, these nuclei appeared more frequently within areas morphologically consistent with neoplastic cell populations. However, as Ki-67 is not tumour-specific, this interpretation represents a biologically plausible assumption rather than a validated distinction between neoplastic and reactive cells. In this cohort, differentiation of neoplastic from reactive cells based solely on histomorphologic criteria (e.g., anisokaryosis, nuclear pleomorphism), particularly on Ki-67-stained sections, was not always unequivocal, especially in tumours with a prominent inflammatory stroma. While strongly Ki-67-positive nuclei were predominantly observed in areas histomorphologically indicative of tumour cell growth, Ki-67-positive leukocytes generally exhibited lower staining intensity. Therefore, high-intensity Ki-67 expression was explored as a potential proxy for tumour-cell enrichment rather than as a tumour-specific marker. This approach was used to assess whether quantification of high-intensity Ki-67 staining could provide a complementary estimate of proliferative activity, independent of staining intensity, and to facilitate practical evaluation in the context of prognostic analyses. Importantly, this strategy does not establish tumour specificity and should be interpreted with caution. Validation using tumour-specific double-labelling approaches (e.g., KIT/Ki-67 co-staining) would be required to confirm that strongly Ki-67-positive nuclei reliably correspond to neoplastic cells. Depending on the size of the tumour section, the time for this fully automated Ki-67 assessment ranged from 9 s to 35:23 min, yielding nuclear counts of 648 and 2.8 million, respectively. The average-sized tumour section (95 mm^2^) with a total nuclear count of 431,999 required 8:23 min. Once analysed, the outputs were exported as tsv files.

#### 2.3.3. Quality Check and Scoring

Once all nuclear labels were defined, each slide was reviewed by a pathologist. Quality scores were given for the level of nuclear segmentation, inflammation and tissue section completeness to assess the reliability of the performed Ki-67 analysis as follows: (i) proportion of tumour tissue with assessable morphology and staining quality, defined as regions exhibiting good cellular/tissue preservation and reliable IHC signal (scoring: 2 > 90%, 1 = 50–90%, 0 < 50%); (ii) inflammatory cell infiltration of the tumour tissue was scored according to the proportion of inflammatory cells relative to tumour cells within the assessed ROI: 2 (negligible, <1%), 1 (mild, 1–10%), or 0 (marked, >10%); nuclear segmentation quality was scored based on the proportion of unlabelled nuclei relative to all nuclei in the ROI: 2 (good, <1% unlabelled), 1 (mildly reduced, 1–10%), or 0 (reduced, >10%). For slide scoring, less than 2 min of slide reviewing were needed. This last step of the workflow is considered essential for a reliable downstream interpretation of the generated Ki-67 expression results.

### 2.4. Clinical Follow-Up

Follow-up data were obtained from referring veterinarians via telephone interviews or an electronic survey. For cases submitted to the German laboratory, telephone follow-up was conducted for a case collection of 68 dogs, yielding outcome data for 39 dogs (response rate: 57%). For cases submitted to the Swiss laboratory, 55 veterinary practices and clinics were contacted by email and invited to complete an electronic survey covering 115 individual cases. Twenty practices returned completed surveys, providing follow-up information for 31 dogs with confirmed mast cell tumours. This corresponds to a practice-level response rate of 36% and a case-level response rate of 27% for the Swiss cases. Follow-up data were available for 68 dogs submitted between 2012 and 2022. For each dog, the collected information included the date of the first and any subsequent (if applicable) MCT biopsy sampling (submitted either to our laboratory or external laboratories), the tumour sample location, the type of therapy administered (surgery, chemotherapy, radiotherapy, prednisolone, other, or none), the response to treatment (yes, only temporary, or no), the occurrence of death (yes/no), the date of death, and the cause of death (MCT-related, unrelated to MCT, or unknown). Based on these data, the age at the first and any following biopsy was calculated. Survival time was defined as the interval between the date of the initial sampling and the date of death, when applicable. For dogs still alive at the time of data collection, survival time was defined as the interval between the initial sampling and the date of data collection. A total of 72 MCTs and corresponding tissue slides were available from 68 dogs, as two dogs had recurrences, one developed a new tumour, and one presented with two concurrent MCTs. The cohort included 25 breeds, including mixed-breed dogs, with a mean age at diagnosis of 9 years (range 2–15 years) and a balanced female-to-male ratio of 1:1.28. Thirty-four of the 68 dogs (50%) were neutered (Supplementary Table S3). Tumour locations were variable, with the toe (39/68; 57%), limb (9/68; 13%), and trunk (8/68; 12%) representing the most common sites. Recurrence was confirmed in twelve dogs. For 62/68 dogs, live status and cause of death were available, with the cause of death determined by the attending clinicians. Data on whether necropsies were performed to confirm the cause of death were not available. MCT recurrence was confirmed in 12 of 68 dogs (18%) and ruled out in 7 of 68 dogs (10%); recurrence status was unavailable for the remaining 49 cases. Most dogs (34/68; 50%) were treated with surgery alone. Chemotherapy was reported in only one case, administered in combination with prednisolone. For 31 of 68 dogs (46%), treatment information was unavailable. At the time of the follow-up, the majority of dogs (36/62; 58%) had died, including 22 deaths attributed to MCT. The median overall survival time was 566 days; when dogs that died from causes other than MCT were censored, the mean survival time was reduced to 225 days.

### 2.5. Statistical Analysis

Statistics were performed using NCSS 2024 Statistical Software version 2024.0.3 (NCSS, LLC. Kaysville, UT, USA). Data normality was assessed using the Shapiro–Wilk test. Statistical significance was set at α = 0.05 for all tests. No correction for multiple testing was applied; *p*-values are reported as exploratory. Continuous Ki-67 metrics, including H-score, proliferation index (PI), positive and strong nuclear densities, and the hotspot Webster index, were compared between two categorical groups—such as sex, castration status, risk breed, Kiupel grading, and affected limb—using the Wilcoxon rank-sum test, as the data were not normally distributed. For comparisons across three categorical groups as defined by the Patnaik grading, the Kruskal–Wallis test was applied. Associations between continuous Ki-67 metrics and other continuous variables, such as age or Webster hotspot counts, were evaluated using Spearman correlation. The relationship between Ki-67 metrics and survival time was assessed with univariable Cox proportional hazards regression models, analysing H-score, PI, and strong nuclear density separately. To illustrate survival differences, Kaplan–Meier curves were generated after dichotomizing each Ki-67 parameter at its median.

## 3. Results

### 3.1. Hotspot Ki-67 Expression

Manual hotspot Ki-67 counts were available for 64 of the 309 studied cases, derived from a previously published set of MCTs of the toe [23]. The mean number of Ki-67-positive cells per hotspot was 24 (range: 1–77; median: 24), close to the prognostic threshold of 23 proposed by Webster et al. in 2007 [14]. For an additional 178 cases of cutaneous MCTs from various anatomical sites, manual hotspot Ki-67 counts were categorized as ≤23 or >23. Across all 242 cases with available hotspot Ki-67 counts, 149 (62%) had counts ≤23, while 93 (38%) had counts >23.

### 3.2. Global Ki-67 Expression

Reliable Ki-67 quantification depends on a low proportion of non-neoplastic cells, as their presence may confound the proliferation index. To account for this potential bias, Ki-67 results were stratified according to tumour inflammation score. The following results are, therefore, reported for all tumours combined and for a subset excluding tumours with the highest levels of inflammation (quality score 0). In addition, output parameters incorporating Ki-67 staining intensity were defined alongside simple Ki-67 positivity. As outlined above, and based on histomorphologic assessment, strongly positive nuclei were considered more likely to represent neoplastic rather than reactive or inflammatory cells and were, therefore, explored as a potentially more specific estimate of tumour cell proliferation. However, since Ki-67 is not tumour-specific, this interpretation should be regarded as an operational assumption rather than a validated distinction between neoplastic and non-neoplastic cells. Across the entire case collection, the majority (186/306 [61%], or 143/204 [70%] after excluding tumours with high inflammation scores) of tumours exhibited low (≤15% PI) Ki-67 labelling, characterized by scattered positive nuclei with weak to strong staining intensity and occasional mild heterogeneity or positive cell clustering. However, a distinct subset of cases (39/306 [13%], or 12/204 [6%] after excluding tumours with high inflammation scores) showed high and diffuse Ki-67 expression, with a proliferation index of ≥30% and often strong staining intensity.

Substantial granulocytic infiltration (eosinophils or neutrophils) was present in 103 out of 309 (33%) cases, and granulocytes typically showed either weak Ki-67 positivity or remained unstained. Given that granulocytes are terminally differentiated cells, their Ki-67 staining was considered nonspecific. Eosinophils often predominated; however, neutrophils were numerous in ulcerated tumours. Granulocytes were usually readily identifiable morphologically, although they were not systematically excluded from the analysis. Therefore, the Ki-67 metrics retrieved from cases with high levels of granulocyte infiltrates (tumour inflammation score 0) were less precise. Occasionally, low numbers of lymphocyte aggregates were present within the tumour tissue, which were excluded from the ROI and not assessed. Other non-neoplastic cells, including histiocytic and reactive fibrovascular stromal cells, showed variable Ki-67 expression and were often difficult to distinguish from tumour cells solely on morphology. Consequently, cases with high inflammatory scores or substantial reactive stroma were considered less reliable for global cell-based quantification because Ki-67 is not specific to neoplastic cells.

The global Ki-67 expression metrics of the studied mast cell tumours (MCTs) are summarized in Table 1. The assessed tumour area ranged from 0.1 to 415 mm^2^ (mean: 96 mm^2^), corresponding to an average 300-fold increase relative to the conventional hotspot area of 0.3125 mm^2^ used in traditional Ki-67 scoring. On average, approximately 670,000 cells were analysed per case, reaching more than 4 million cells in the largest and most cellular tumour sections. Overall, the proportion of Ki-67-positive cells was low, with a mean proliferation index (PI) of 14%. Exclusion of all cases with the highest inflammatory scores reduced the mean PI by 3%. Mean H-scores were likewise low (mean: 26), decreasing by 12% to 21 following exclusions of tumours with marked inflammation. This reduction in Ki-67 metrics after removal of highly inflamed cases was expected, given the observed nonspecific Ki-67 staining of leukocytes in these tumours. Positive cell density (i.e., positive nuclear counts per mm2 of tumour tissue) was roughly 1000 on average, with up to a 9-fold increase in tumours with the highest Ki-67 expression. Based on histomorphology, strongly stained nuclei were considered tumour cell-specific, whereas weak to moderate staining was also observed in inflammatory and reactive tissue cells. The parameter strong positive cell density was, therefore, considered the most reliable for measuring tumour-specific Ki-67 expression. The mean was 346 strongly stained (tumour) cells per mm^2^ of tissue. Excluding cases with high inflammation scores led to a small reduction in the calculated density to 305/mm^2^, a 12% decrease.

### 3.3. Correlation of Global Ki-67 Metrics with Other Case Parameters

The global Ki-67 expression was evaluated for any correlation with the provided case parameters and defined tumour characteristics (Table 2).

#### 3.3.1. Signalment

The mean age of the studied dogs was 8 years (range, 0–21 years). Female dogs were slightly overrepresented (163/297; 55%). Approximately half of the female dogs were neutered (85/163; 52%), whereas male dogs were more frequently intact than castrated (81/134; 60%). However, castration status was unreliable due to incomplete reporting and unknown age at castration.

A total of 65 breeds were represented (Supplementary Table S1). Mixed-breed dogs were most common (16%), followed by Labrador Retrievers (14%) and French Bulldogs (9%). Breeds previously reported to be at increased risk for mast cell tumour development [3,26] accounted for 56% of the cohort, whereas breeds associated with high-grade MCTs comprised only 5% [27]. No correlation was observed between global Ki-67 expression (measured using PI, H-score, and strongly positive cell density) and breed, age, sex, or neuter status (Table 2). Global Ki-67 assessment revealed a significantly increased proliferation rate in tumours from the hind compared with the forelimb (mean H-score 15 vs. 8; mean PI 9 vs. 5). This effect was only present after exclusion of tumours with high levels of inflammation (assigned quality score 0). Highly inflamed tumours compromise reliable Ki-67 quantification because of the substantial presence of non-tumour cells that cannot be excluded from the analysis. Consequently, the inclusion of tumours with significant inflammation may obscure true differences in Ki-67 expression between hind- and forelimb tumours. Alternatively, the observed differences in Ki-67 expression may be associated with the degree of tumour inflammation rather than tumour location. Notably, this hind–forelimb difference in Ki-67 expression was not detected using manual hotspot-based Ki-67 assessment, irrespective of whether highly inflamed tumours were excluded.

#### 3.3.2. Manual Hotspot Ki-67

For the first two case series, the hotspot Ki-67 Webster index was available and was defined as high (>23) or low (≤23). The global Ki-67 metrics correlated strongly with the hotspot Webster category (high vs. low), with mean H-scores, PI, and strong nuclear densities of 40 vs. 24, 20% vs. 14%, and 634/mm^2^ vs. 235/mm^2^, respectively (Table 2). Spearman correlation analysis confirmed moderate correlations between the hotspot Ki-67 Webster category (high vs. low) and the global H-score (ρ = 0.58) and PI (ρ = 0.51), which were slightly reduced after exclusion of tumours with high inflammation score (ρ = 0.50 and ρ = 0.43, respectively). The global Ki-67 strong nuclear density showed a strong correlation with the hotspot category (ρ = 0.76), with a mild reduction after excluding tumours with significant inflammation (ρ = 0.71). These findings confirm that the selected hotspot is proportional to the level of cell proliferation throughout the entire tumour section. To determine how much more proliferative the hotspot region was compared with the tumour overall, hotspot-positive cell density was calculated by dividing hotspot-positive cell counts by the assumed hotspot area of 0.3125 mm^2^ (as described by Webster et al. [14]). This hotspot density was then compared with the global density of strongly positive Ki-67-stained cells (cells/mm^2^). Strongly positive cells, rather than all positive cells, were selected as the reference parameter because weak and moderate staining was considered nonspecific in a substantial proportion of tumours and was frequently associated with leukocytes rather than neoplastic cells. The proliferation factor was calculated as the ratio of global to hotspot density, representing the fold difference between the overall and hotspot Ki-67 burdens. On average, the manual Ki-67 positive cell density was twice (all tumours combined) to three times (tumours with high inflammation score excluded) as high in the tumour hotspot compared with the whole tissue section, with a range of 38-fold.

#### 3.3.3. Traditional Tumour Grading Systems

Global Ki-67 was not associated with tumour grade as defined by the two traditional tumour grading systems by Patnaik and Kiupel. This finding was unexpected, given that both tumour grading systems include histomorphologic criteria indicating increased cell proliferation, such as mitotic count and nuclear atypia. The lack of correlation between tumour cell proliferation and established tumour grading schemes should be interpreted with caution, as only MCTs of the toe were evaluated, which represent a specific tumour subcategory.

### 3.4. Global Ki-67 Expression as Prognostic Marker

**Figure 3:**
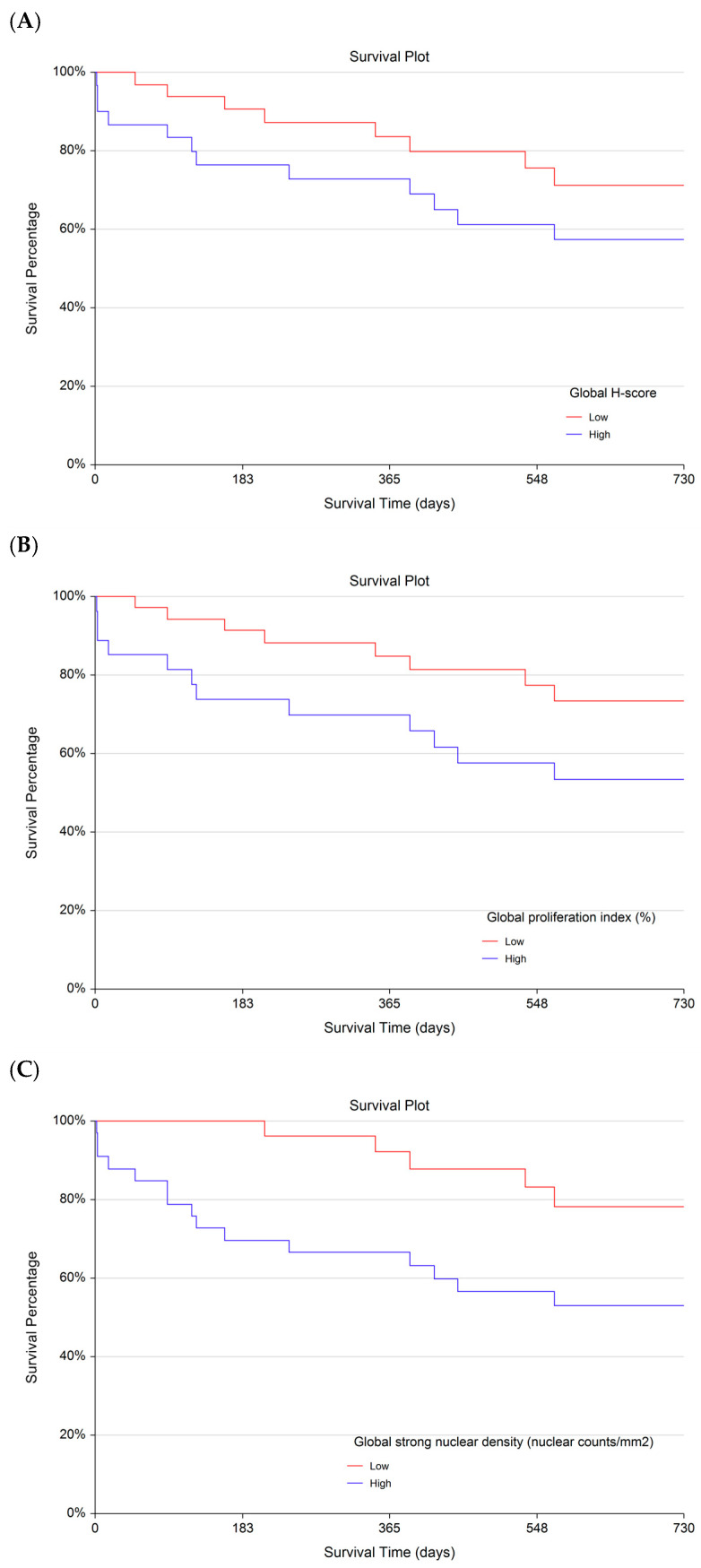
Kaplan–Meier survival analyses of 68 dogs with confirmed cutaneous mast cell tumours, illustrating overall survival up to 730 days (2 years). Survival curves are shown for three global, digitally derived Ki-67 parameters: (**A**) Ki-67 H-score, (**B**) Ki-67 proliferation index (PI), and (**C**) density of strongly Ki-67-positive nuclei. Tumours were dichotomised at the median value of each parameter into Ki-67-high (red) and Ki-67-low (blue) groups. Survival time was defined as the interval between initial tumour diagnosis and death or censoring.

Follow-up data were available for 68 dogs, allowing an exploratory analysis of global Ki-67 metrics as potential predictors of survival (Supplementary Table S3). For visualization purposes, tumours were dichotomized into Ki-67-high and Ki-67-low groups using the respective median values (H-score: 8; PI: 5%; strong nuclear density: 72). Across all three parameters, Kaplan–Meier curves showed visual separation between groups, with an estimated 2-year survival of <60% in Ki-67-high tumours compared with <80% in Ki-67-low tumours (Figure 3A–C). Cox proportional hazards regression identified statistically significant associations between each Ki-67 parameter and survival (*p* < 0.05 for all; Table 3). However, hazard ratios ranged from 0.68 to 1.24, indicating small effect sizes per unit change and, for global strong nuclear density, an effect estimate close to 1. These findings suggest that, although median-based dichotomisation produced visually separable survival curves, the magnitude of the prognostic effect in this cohort was limited. The median thresholds were applied solely for graphical illustration and do not represent clinically validated cut-offs.

## 4. Discussion

This study demonstrates the feasibility and potential clinical utility of global, semi-automated Ki-67 assessment in canine MCTs using a commercial digital pathology software, aiming to enable more reliable and precise prognostication in routine diagnostic practice. Across 309 cases from two different pathology diagnostic institutions, global Ki-67 metrics aligned well with conventional manual hotspot counts (Spearman ρ = 0.76 for strong nuclear density). Exploratory survival analysis in a subset of 68 cases demonstrated statistically significant associations between global Ki-67 parameters and survival outcomes. However, the observed effect sizes were modest, and hazard ratios—particularly for global strong nuclear density—were close to unity, indicating limited prognostic impact within this cohort. These findings, therefore, suggest statistical associations rather than strong or clinically actionable prognostic effects. Notably, the workflow required frequent, and sometimes substantial, manual refinement of the region of interest due to tumour heterogeneity, ill-defined tumour boundaries, inflammation, and tissue artefacts. This step proved to be the principal time-limiting component of the analysis and currently limits the immediate feasibility of fully automated, high-throughput implementation in routine diagnostics. Altogether, these findings support global Ki-67 assessment as a promising standardised approach for canine MCT prognostication, whilst highlighting practical challenges that must be addressed for routine clinical implementation.

Manual quantitative assessment of IHC markers such as Ki-67 has traditionally been restricted to small tissue regions to remain feasible. The area with the highest expression of the marker of interest—referred to as the hotspot—has been defined as the most relevant region of interest (ROI). In this context, “highest expression” is typically determined by either absolute or relative counts of positively stained cells. The main limitations of such manual approaches, including high interobserver variability and substantial time requirements, are well recognized [28,29,30]. The Ki-67 proliferative index serves as a pivotal prognostic marker in many cancers, including breast and prostate cancer, and neuroendocrine (NE) tumours [25,28,29,30,31,32,33,34]. For digestive system NE tumours, the Ki-67 index is considered the most reliable prognostic factor, and the WHO strongly recommends its precise assessment [31]. A recent survey confirmed considerable variability among pathologists regarding Ki-67 counting methods and the definition of positive staining [32]. With the evolution of digital pathology, the limitations of manual hotspot-based approaches have become even more apparent. This is because global assessment with automated hotspot definition is now feasible, and IHC signals and their corresponding quantitative parameters cannot remain merely descriptive; they must be explicitly defined when using digital pathology software [33]. While it is well established that hotspot size influences Ki-67 results, it is also important to recognize that its location and geometric shape can significantly affect the Ki-67 index [35].

Extending Ki-67 assessment from manual, hotspot-based evaluation to global, automated analysis of entire tumour sections has substantial potential to improve both diagnostic and prognostic accuracy. The main advantages include the following: (i) markedly increased objectivity, consistency, and precision; (ii) a more comprehensive representation of the entire tumour; (iii) the ability to quantify spatial heterogeneity and (iv) reduced labour and time demands for pathologists by replacing manual counting with automated analysis. Recent evidence demonstrates that Ki-67 heterogeneity and global assessment-derived proliferation indices (PI) carry significant prognostic value. Maximum Ki-67 PI obtained by global assessment was identified as the sole independent predictor of overall survival in enteropancreatic NE tumours [36]. Studies further show that global mapping-based hotspot selection improves the identification of higher-grade tumours and that digital image analysis (DIA) allows accurate grading with greater time efficiency than manual counting [37]. DIA-derived spatial heterogeneity metrics directly relate to tumour classification and grading and improve discrimination of high-grade neuroendocrine neoplasms [38,39]. The International Ki-67 in Breast Cancer Working Group (IKWG) recommends calculating Ki-67 globally across the tumour area, and these recommendations have been integrated into many international breast cancer guidelines. Best practices for meningioma prognostication include global Ki-67 assessment across multiple blocks [40]. Recently, the Ki-67 global score was reported to be superior to Ki-67 hotspot indices and mitotic count for prognosis in a study of canine anal sac adenocarcinomas [41]. In a recent canine survey, the global evaluation of Ki-67 expression revealed variation in spatial proliferation patterns across different neoplasms, including cutaneous MCT [42]. Ki-67 is a well-established prognostic marker for canine mast cell tumours (MCT) [20]; however, global Ki-67 expression levels and their prognostic relevance have not yet been reported, to the authors’ knowledge.

Our findings underscore several practical considerations for implementing global Ki-67 assessment in canine MCT. With the increasing availability of user-friendly commercial digital pathology platforms, global quantification of Ki-67 and other IHC markers warrants broader exploration and standardization. Because global analysis captures a substantially larger portion of the tumour than traditional hotspot-based methods, it offers a more representative measure of proliferative activity. It may provide a stronger foundation for future guidelines. However, fully automated workflows remain limited by the pronounced histomorphologic heterogeneity typical of MCT. In many cases, the ROI requires manual refinement to exclude non-neoplastic tissue or artefacts, a process that has taken up to 30 min per slide in our slide collection. Once the ROI is defined, the subsequent steps—automated nuclear detection, classification, and quantitative output—are rapid and consistent. Expert review by pathologists also remains essential to ensure the reliability of global analyses. Several methodological considerations inherent to Ki-67 assessment warrant acknowledgment and apply to both conventional and automated approaches. Even with standardized automated platforms, Ki-67 immunohistochemistry remains technically challenging, with well-documented interlaboratory variability attributed to factors including tissue processing, fixation protocols, and reagent selection [43,44]. Beyond technical aspects, automated approaches currently cannot reliably distinguish neoplastic from non-neoplastic Ki-67 expression, and proliferating inflammatory and reactive stromal cells may contribute to the overall proliferation index and substantially distort automated measurements [45,46]. While manual ROI refinement addresses some of these challenges, the biological interpretation of Ki-67 indices should consider the potential influence of inflammatory and stromal components in the tumour microenvironment [47]. Although Ki-67 positivity in tumour cells, irrespective of staining intensity, ideally needs to be recorded, in our case, nuclei with intense Ki-67 staining were more frequent in areas histomorphologically consistent with neoplastic growth, particularly in cases showing substantial Ki-67 expression in reactive non-tumour cells. Emphasizing strong Ki-67 positivity may, therefore, enrich for tumour-cell–associated proliferation; however, this approach should be interpreted cautiously, as weaker staining can also represent proliferating neoplastic cells and staining intensity alone does not establish tumour specificity. Further validation using tumour-specific double-labelling strategies would be required to confirm the reliability of this distinction. In tumours with extensive inflammation, reactive stroma, or poorly defined boundaries, non-neoplastic Ki-67-positive cells can substantially distort automated measurements. At present, no robust method exists to automatically distinguish and exclude non-tumour cells in canine MCTs, and digital Ki-67 assessment may, therefore, be unreliable in selected cases of highly inflammatory profiles. This limitation also applies to manual Ki-67 evaluation in MCTs and other neoplasms; however, uncertainty in discriminating between tumour and non-tumour cells is typically neither recorded nor adequately described in both routine clinical practice and scientific publications that rely on manually assessed Ki-67. A mast cell-specific marker (e.g., KIT) would need to be combined with Ki-67 to enable tumour-specific proliferation assessment, analogous to established approaches in breast cancer where cytokeratin is combined with Ki-67 [48,49]. Until this limitation is addressed, automated Ki-67 assessment should be considered unreliable and interpreted with caution in tumours with substantial non-tumour cell populations.

Ki-67 indices have been shown to correlate with tumour grade under both the Patnaik and Kiupel grading systems [16,50]. This trend was also observed in our case collection, but did not reach statistical significance. This is most likely attributable to the limited availability of tumour grade data, which were confined to a subset of toe MCTs, and to a marked imbalance between Kiupel low- and high-grade tumours (58 vs. 8 cases). Nevertheless, assessing the relationship between proliferative activity and histological grade remains essential, as integrating Ki-67-based metrics with tumour grading may improve prognostic stratification beyond either parameter alone.

Accurate survival prediction in canine mast cell tumours remains challenging, particularly for intermediate-grade lesions with highly variable clinical behaviour. Our findings support the concept that global, whole-slide Ki-67 metrics provide a more comprehensive and reproducible assessment of tumour proliferation than conventional hotspot-based manual approaches, primarily by capturing intratumoral heterogeneity. However, the exploratory survival analysis performed in this study demonstrated only modest effect sizes, and no clinically meaningful Ki-67 thresholds can be derived from the present data. Importantly, median-based dichotomisation was applied solely to facilitate graphical visualization of survival differences and does not imply the existence of biologically or clinically validated cut-off values. Establishing clinically relevant reference ranges and prognostic thresholds will require larger, prospectively designed studies with standardized global Ki-67 quantification. The survival analysis in this cohort was further limited by the relatively small sample size and heterogeneity in dog breeds and tumour locations, which restricts statistical power and generalizability. In addition, follow-up data were retrospective, the cause of death was based on clinician assessment without systematic necropsy confirmation, and information regarding tumour recurrence, concurrent neoplasia, and adjuvant chemotherapy was incomplete for a substantial proportion of cases. These variables may independently influence survival and should be considered potential confounders when interpreting the observed associations between Ki-67 expression and outcome. Case selection and study design would require optimization to enable more robust survival modelling and the reliable definition of prognostic Ki-67 thresholds. Importantly, Ki-67 alone is unlikely to be sufficient for robust prognostication. Instead, proliferation metrics should be integrated with established parameters such as histomorphologic tumour grading, KIT mutation status, and KIT immunohistochemical staining patterns. Beyond these markers, future studies should explore additional molecular or microenvironmental biomarkers that may further refine risk stratification. In this context, digital pathology offers a significant advantage by enabling objective, reproducible, and scalable analyses. Analogous to Ki-67, automated whole-slide evaluation of KIT immunohistochemistry may reduce observer bias and improve consistency compared with manual pattern classification. User-friendly digital pathology platforms are now commercially available and enable such analyses in routine diagnostic settings, with feasible hardware, software, and pathologist-training requirements. Together, multiparametric digital histopathologic approaches hold promise for advancing prognostic precision in canine MCTs.

## 5. Conclusions

In the era of digitalization, automated, objective assessment of Ki-67 and other tissue biomarkers should become the new standard for tumour evaluation. Commercial digital pathology software is now widely available and enables rapid, reproducible analysis across entire tumour tissue sections. However, accurately defining the region of interest often requires substantial manual refinement due to tumour heterogeneity and variable tissue and staining quality. While global Ki-67 assessment provides a promising and objective analytical framework, further improvements in automated tumour segmentation will be essential before this approach can be realistically implemented as a routine diagnostic standard. Additionally, Ki-67 is not specific to tumour cells, which can render measurements unreliable in tumours with significant inflammation, reactive stroma, or poorly defined boundaries. Despite these limitations, global digital assessment provides a more representative and standardized approach to evaluating proliferation and other relevant tissue markers, can improve prognostic precision in canine mast cell tumours, and highlights challenges that need to be addressed in future studies.

## Figures and Tables

**Table 1:** Overview output derived from digital analysis of global Ki-67 expression in canine mast cell tumours (MCT). For the assessed tissue area, assessed nuclei count, and Ki-67 metrics, mean values are indicated with ranges and standard deviations (SD) in brackets.

Parameter	Output			Fold Change After *
**Count all slides**	309			
Count all slides *	206			
**Assessed tissue area_mm^2^**	96 (0–415; 89)			
**Count assessed nuclei per slide (10^5^)**	6.7 (0.01–43.1; 7.2)			
Slide quality score ** **Tumour tissue section** **Nuclear segmentation** **Inflammation**	0 15 (5%) 1 (0%) 103 (33%)	1 17 (6%) 3 (1%) 72 (23%)	2 278 (90%) 305 (99%) 135 (44%)	
**H-score (0–300)**	26 (0–138; 25)			
H-score (0–300) *	21 (0–138; 22)			−1.24
**Proliferation Index (%)**	14 (0–58; 13)			
Proliferation Index (%) *	11 (0–57; 11)			−1.27
**Count positive nuclei per mm^2^**	1056 (2–9178; 1187)			
Count positive nuclei per mm^2^ *	845 (10–5880; 949)			−1.25
**Count strong nuclei per mm^2^**	346 (1–5234; 521)			
Count strong nuclei per mm^2^ *	305 (1–3663; 456)			−1.13

* Cases with high levels of inflammation (quality score 0) were excluded. ** Quality score for tumour tissue section refers to the proportion of tumour tissue with assessable, good quality morphology and staining. Scoring: 2 > 90%, 1 = 50–90%, 0 < 50%); nuclear segmentation quality was scored based on the proportion of unlabelled nuclei as 2 (good, <1% unlabelled), 1 (mildly reduced, 1–10%), or 0 (reduced, >10%); tumour inflammation was scored according to the proportion of inflammatory cells relative to tumour cells as 2 (negligible, <1%), 1 (mild, 1–10%), or 0 (marked, >10%).

**Table 2:** Correlation of signalment, manual hotspot, and digital global Ki-67 metrics in canine mast cell tumours. *p*-values are indicated for significant correlations between specific parameters. Abbreviations: F: Female; M: Male; NA: not available or applicable; NP: not performed; ns: not significant; PI: proliferation index (%).

Ki-67 Metrics			Manual Hotspot		Digital Global					
Parameter	Slide		Hotspot Ki-67 Count		H-Score		PI		Count Strong Nuclei per mm^2^	
		#		#		#		#		#
Signalment	N	N								
Age	300 NA (9)	204	ns	ns	ns	ns	ns	ns	ns	ns
Sex	F (165) M (135) NA (9)	F (116) M (86)	ns	ns	ns	ns	ns	ns	ns	ns
Castration status	entire (162) neutered (143) NA (4)	entire (102) neutered (102)	ns	ns	ns	ns	ns	ns	ns	ns
Breed (high incidence) ^β^	at risk (167) non-risk (132) NA (10)	at risk (113) non-risk (87) NA (4)	ns	ns	ns	ns	ns	ns	ns	ns
Breed (high-grade tumour) ^‡^	at risk (15) non-risk (284) NA (10)	at risk (7) non-risk (193) NA (4)	NP ^+^	NP ^+^	ns	ns	ns	ns	ns	ns
Affected limb (fore vs. hind)	fore (35) hind (18) NA (256)	fore (21) hind (11) NA (172)	ns	ns	ns	*	ns	*	ns	ns
Affected limb (right vs. left)	right (18) left (35) NA (256)	right (10) left (22) NA (172)	ns	ns	ns	ns	ns	ns	ns	ns
Traditional scores	N	N								
Hotspot Ki-67 count ^&^	64 NA (245)	38 NA (166)	NA	NA	****	*	****	**	****	****
Hotspot Ki-67 count ^&^ (categorical)	≤23 (149) >23 (93) NA (67)	≤23 (95) >23 (59) NA (50)	NA	NA	****	****	****	****	****	****
Grading Patnaik	I (14); II (45); III (7) NA (243)	I (7); II (29); III (4) NA (164)	ns	ns	ns	ns	ns	ns	ns	ns
Grading Kiupel	low (58) high (8) NA (243)	low (36) high (4) NA (164)	ns	ns	ns	ns	ns	ns	ns	ns

# after exclusion of cases with high levels of inflammation (quality score 0). ^β^ breeds with increased risk of MCT incidence, as defined by [3,26]: bulldog-related breeds, pug, Golden retriever, Labrador retriever, Shar Pei, Rhodesian ridgeback, Vizsla, Bernese Mountain dog, Beagle, and Weimaraner. ^‡^ breeds with known risk for high-grade MCTs, as defined by [27]: American Staffordshire terrier, Dachshund, German shepherd, Shar Pei, and Weimaraner. ^&^ as defined by Webster et al. 2007 [14]. ^+^ Hotspot Ki-67 values are not available for cases with increased risk of high-grade tumours. *p* values are indicated by asterisks: *p* < 0.05 (*), *p* < 0.01 (**), *p* < 0.0001 (****).

**Table 3:** Cox regression-derived hazard ratios (HR), 95% confidence intervals (CI), and *p*-values are presented for the three global Ki-67 parameters to assess their relevance as prognostic tumour markers.

Parameter	HR	95% CI	*p*-Value
**Global Ki-67 H-score**	1.24	1.08–1.41	0.0019
**Global Proliferation Index (%)**	0.68	0.51–0.91	0.0089
**Global strong nuclear density**	1.0	1.00–1.00	0.0011

## Data Availability

The original contributions presented in this study are included in the article. Further inquiries can be directed to the corresponding authors.

## Supplementary Materials

The following supporting information can be downloaded at https://www.mdpi.com/article/10.3390/vetsci13020198/s1, Table S1. Demographics of the studied cases and provided case data. Table S2. Comparison of Immunohistochemical Protocols of the two participating institutions. Table S3. Overview of the 68 dogs investigated with available follow-up data.

## Abbreviations

The following abbreviations are used in this manuscript:

AgNORs	Argyrophilic nucleolus organizer regions
AI	Artificial intelligence
DAB	3,3′-diaminobenzidine
DL	Deep learning
FFPE	Formalin-fixed and paraffin-embedded
H-score	Histology score
HDAB	Haematoxylin-DAB
IHC	Immunohistochemistry
MCT	Mast cell tumour
NE	Neuroendocrine
NP	Not performed
Ns	Not significant
PI	Proliferation index
ROI	Region of interest
WHO	World Health Organization
WSI	Whole-slide image
