# Supplementary material for: Revisiting Ki-67 Assessment in Canine Mast Cell Tumours: From Manual Hotspot to Automated Global Analysis
Source: Vet Sci. 2026 Feb 18;13(2):198. doi: 10.3390/vetsci13020198 (PMC12945039; doi:10.3390/vetsci13020198)
Supplement: Supplementary file 1 [file vetsci-13-00198-s001.zip › vetsci-4101058-supplementary.pdf]

Table S1. Demographics of the studied cases and provided case data. *As defined by Webster et al. in 2007 [14]. Abbreviations: MCT: mast cell tumour; N: total sample size; SD: standard deviation.

Signalment	N (TOTAL N=309)	(%)
**Breed**		
**a) Top three**	Mix (49)	16
	Labrador retriever (43)	14
	French Bulldog (27)	9
**b) Represented breeds**	65	
**Sex**		
**Female neutered**	88	28
**Female intact**	77	25
**Male neutered**	53	17
**Male intact**	80	26
**Unknown**	11	4
**Age (years) mean (range; SD)**	8 (0-21; 3)	
**Tumour location**		
**Limb**	11	11
**Trunk**	7	7
**Head/neck**	3	3
**Inguinal**	2	2
**Perineum**	1	1
**Genital**	1	1
**Digit**	68	71
**Tail**	3	3
**Tumour grade Patnaik**		
**Grade 1**	14	21
**Grade 2**	45	68
**Grade 3**	7	11
**Tumour grade Kiupel**		
**Low grade**	58	88
**High grade**	8	12
**Manual hotspot Ki67* mean (range; SD)**	24 (1-77; 18)	
**Manual hotspot Ki67***		
**Low (≤23)**	149	62
**High (>23)**	93	38
**Clinical follow-up**		
**Alive**	26	42
**Dead of disease**	22	36
**Dead of unrelated disease**	14	23
**Mean survival time (days) mean (range; SD)**	825 (0-2960; 782)	

Table S2. Comparison of Immunohistochemical Protocols of the two participating institutions. Abbreviations: IHC = immunohistochemistry; DAB = 3,3'-diaminobenzidine; RT = room temperature.

Parameter	Case Series Germany	Case Series Switzerland
**Number of cases**	248/309 (80%)	61/309 (20%)
**Tissue section thickness**	2 μm	2-3 μm
**Slide type**	Coated slides	Positively charged slides (Color Frosted Plus, Biosystems)
**Drying**	Overnight at 58°C	35 min at 60°C
**Dewaxing**	Standard protocol	Bond Dewax solution (Leica Biosystems)
**Instrumentation**	Manual protocol	Bond-III immunostainer (Leica Biosystems)
**Antigen retrieval buffer**	EDTA buffer (Zytomed System GmbH)	Bond epitope retrieval solution 2 (Leica Biosystems)
**Antigen retrieval pH**	pH 9.0	pH 9.0
**Antigen retrieval temperature**	96°C	95°C
**Antigen retrieval duration**	25 min	20 min
**Primary antibody**	Monoclonal anti-mouse MIB-1 (Dako, #M7240)	Monoclonal anti-mouse MIB-1 (Dako, #M7240)
**Primary antibody dilution**	1:200	1:50
**Primary antibody incubation**	60 min at room temperature	15 min at room temperature
**Blocking**	Not specified	Protein blocking solution, 10 min at RT
**Detection system**	ZytoChem Plus HRP Polymer Kit	Bond Polymer Refine Detection Kit (Leica Biosystems)
**Secondary antibody/Polymer**	Part of polymer kit	8 min each (supplemented with 2% dog serum)
**Dog serum addition**	No	Yes (2% in secondary and polymer)
**Chromogen**	DAB	DAB/H_2O_2
**Chromogenic development**	Not specified	10 min

**Counterstain**	Mayer's haematoxylin	Haematoxylin
**Positive control**	Canine lymph node	External (human epidermis) and internal (canine epidermis)
**Negative control**	Primary antibody replaced with buffer	Primary antibody replaced with buffer

Table S3. Overview of the 68 dogs investigated with available follow-up data. Abbreviations: CTx: chemotherapy; DNOD: dead of unrelated disease; DOD: dead of disease; dx: diagnosis; F: female entire; FN: female neutered; M: male entire; MN: male neutered; NA: not available; NS: not specified; PI: proliferation index (%); Pred: prednisolone; Rec: recurrence; RTx: radiation therapy; ST: survival time; Sx: surgery; Tx: treatment; 2nd T: second tumour (MCT). * ST is defined as time period between the sampling of the assessed tumour tissue and end of follow up or death. & Confirmed lymph node metastasis. + Suspected lymph node metastasis.

**Signalment**											**Global Ki-67 metrics**			
**Case**	**Year of submission / initial diagnosis**	**Sex**	**Breed**	**Age (years) at first dx**	**Tumour location**	**Status**	**Rec**	**Other tumours**	**Tx**	**ST (days)***	**H-score**	**PI (%)**	**count strong nuclei per mm²**	**tumour inflammation (0: high 1: intermediate 2: low)**
**1**	2012	M	Jack Russell terrier	13	Trunk	DNOD	yes		Sx	1252	1	1	16	0
**2**	2012	_N	Bernese Mountain Dog	5	Trunk	DOD	yes		Sx	533	1	1	1	1
**3**	2012	F	NA	14	NA	NA	NA		NA	569	1	0	11	1
**4&**	2012	M	Boxer	8	Tail	DOD	yes		Sx	569	5	2	44	2
**4 (2nd T)**									Sx	569	16	7	355	0
**4 (Rec)**	2014								Sx	50	7	3	103	0
**5**	2013	F	Golden retriever	14	NA	NA	NA		NA	2960	3	2	11	1
**6**	2013	FN	Bernese Mountain Dog	11	Head/neck	DOD	NA		Sx	2	93	38	2110	2
**7**	2013	_N	Labrador retriever	6	Trunk	DNOD	NA		Sx	2960	9	4	104	0
**8**	2015	MN	Labrador retriever	7	Trunk	DNOD	no		Sx	1923	17	8	236	2
**9**	2015	FN	Mix	12	Trunk	DNOD	NA		Sx	122	2	1	5	2
**10**	2015	MN	Mix	8	Inguinal	ALIVE	NA		Sx	2957	2	1	19	2
**11**	2015	FN	Catalan Sheepdog	11	Trunk	DOD	no		Sx	1438	1	1	14	2
**12**	2018	MN	Labrador retriever	5	Head/neck	ALIVE	yes		Sx/RTx	2093	0	0	1	0
**13**	2018	M	Pug	9	Limb	DNOD	no		Sx	1534	16	7	335	1

**14**	2018	F	Mix	7	Limb	ALIVE	NA		Sx	2013	2	1	21	2
**15**	2018	MN	French Bulldog	9	Limb	DNOD	no		Sx	1434	3	2	43	1
**16**	2018	M	Malinois	9	Limb	DNOD	NA		Sx	1645	1	1	28	2
**17**	2018	MN	Labrador retriever	9	Genital	DOD	NA		Sx	1596	0	0	4	1
**18**	2018	MN	Bernese Mountain Dog	7	Trunk	DNOD	NA	Yes (eye lid melanocytoma)	Sx	232	3	2	21	2
**19**[&]	2018	FN	Mix	8	Toe	DOD	NA		Sx	161	6	3	77	2
**20**[+]	2018	M	Labrador retriever	11	Trunk	DOD	NA		Sx	347	1	1	7	2
**21**	2018	MN	Labrador retriever	5	Head/neck	ALIVE	NA		Sx/RTx	2136	6	3	170	2
**22**	2019	FN	Malinois	11	Limb	ALIVE	NA		Sx	1736	1	0	7	1
**23**	2019	FN	French Bulldog	5	Limb	ALIVE	no		Sx	1716	10	5	99	1
**24**	2019	FN	French Bulldog	7	Inguinal	ALIVE	no		Sx	1597	50	22	1439	2
**25**	2020	FN	Do-Khy	11	Limb	DOD	NA		Pred	17	37	16	427	1
**26**	2020	MN	French Bulldog	6	Head/neck	DOD	NA		Sx/Pred	126	17	8	372	0
**27**	2021	M	Yorkshire terrier	15	Inguinal	DOD	NA		Pred	3	68	29	859	2
**27 (2nd T)**					Limb					3	42	18	928	0
**28**	2021	FN	Boston terrier	6	Perineum	ALIVE	NA		Sx	920	2	1	20	2
**29**	2021	FN	Golden retriever	4	Limb	ALIVE	yes		Sx	786	5	2	36	2
**29 (Rec)**	2022									521	7	4	76	1
**30**	2022	M	Bull terrier	5	Limb	ALIVE	NA		Sx	562	8	4	59	1
**31**	2023	M	German pinscher	12	Toe	ALIVE	NA		Sx	196	9	4	208	1
**32**	2014	F	Mix	8	Toe	DOD	yes		NA	240	20.1	12.7	213	0
**33**	2015	M	French Bulldog	5	Toe	DNOD	NA		NA	870	3.9	2.6	18	0
**34**	2016	F	Puli	10	Toe	DNOD	NA	Yes (NS)	NA	730	16.2	9.2	160	2
**35**	2016	M	German Sheperd Dog	8	Toe	NA	NA		NA	60	14	8.8	110	1
**36**	2017	FN	Labrador retriever	4	Toe	ALIVE	NA		NA	1470	14.2	12.2	11	2
**37**	2017	M	Bullmastiff	8	Toe	DOD	NA		NA	30	20.3	16.8	28	0

**38**	2018	F	English Toy Terrier	9	Toe	ALIVE	NA		NA	1770	8	3.9	217	2	
**39**	2018	F	Labrador retriever	11	Toe	NA	NA		Sx	330	25.1	19.5	34	0	
**40**	2018	M	Havanese	11	Toe	ALIVE	NA		NA	1530	0.9	0.7	2	1	
**41**	2018	FN	Beagle	11	Toe	DOD	yes		NA	450	22.7	10	451	0	
**42**	2018	M	Jack Russell terrier	11	Toe	DNOD	NA		NA	690	4.8	3.3	23	2	
**43**	2018	M	Labrador retriever	10	Toe	ALIVE	NA		NA	1470	12.6	6.7	83	1	
**44**	2018	MN	Mix	2	Toe	NA	NA		NA	180	7.1	4.6	35	2	
**45**	2019	F	Cocker Spaniel	12	Toe	DOD	yes		NA	210	3.7	2.1	52	2	
**46**	2019	F	Mix	9	Toe	DOD	yes		NA	90	89.7	42.1	1083	0	
**47**	2019	FN	French Bulldog	7	Toe	ALIVE	NA		NA	1290	29	14.5	730	0	
**48**	2019	FN	French Bulldog	4	Toe	DOD	yes		Pred/C Tx	390	34.7	16.4	556	1	
**49**	2019	F	Briard	11	Toe	DOD	yes		NA	90	5.4	2.8	96	2	
**50**	2019	FN	Maltese	10	Toe	ALIVE	NA		NA	1140	49.5	28.4	245	0	
**51**	2019	M	Golden retriever	3	Toe	ALIVE	NA		NA	960	49.3	31.7	388	0	
**52**	2019	MN	Golden retriever	9	Toe	DNOD	NA		NA	0	24.4	15.3	83	0	
**53**	2019	MN	Mix	9	Toe	DOD	NA		NA	0	9	5.1	69	2	
**54**	2020	M	Pug	4	Toe	ALIVE	NA		NA	1020	42.1	23.2	263	0	
**55**	2020	M	Pug	5	Toe	ALIVE	NA		NA	360	7.5	4.6	67	2	
**56**	2020	M	Bracke	15	Toe	DOD	NA		NA	420	67	34.6	1079	0	
**57**	2020	F	Mix	8	Toe	ALIVE	NA		NA	870	22.2	14.2	142	0	
**58**	2020	M	Pug	5	Toe	DNOD	no	Yes (spleen, intestine NS)	NA	570	5.4	3.3	28	0	
**59**	2021	M	Yorkshire terrier	3	Toe	ALIVE	NA		NA	570	30.8	18.4	433	2	
**60**	2021	F	Labrador retriever	10	Toe	NA	NA		NA	360	1.5	1.1	8	0	
**61**	2021	MN	French Bulldog	12	Toe	DOD	NA		Sx	390	6.3	4.5	19	2	
**62**	2021	MN	Yorkshire terrier	11	Toe	DNOD	NA	Yes (anal sac carcinoma)	Sx	300	32.8	19.6	212	0	

**63**	2021	MN	Golden retriever	10	Toe	DOD	yes		NA	120	13.5	9	139	0
**64**	2021	M	Labrador retriever	10	Toe	ALIVE	NA		Sx	390	1	0.7	5	2
**65**	2022	FN	Mix	9	Toe	ALIVE	NA		Sx	300	6.9	4.5	36	2
**66**	2022	FN	Golden retriever	12	Toe	DOD	NA		Sx	0	15.7	10.3	91	1
**67**	2022	M	Pug	8	Toe	ALIVE	NA		Sx	210	1.3	0.9	4	1
**68**	2022	MN	Mix	7	Toe	ALIVE	NA		NA	90	15	10.8	57	1
